# Unintended consequences of co-payment regulations in Belgium: the case of atorvastatin

**DOI:** 10.1186/2052-3211-8-S1-P1

**Published:** 2015-10-05

**Authors:** Jessica Fraeyman, Hans De Loof, Guido Van Hal, Guido De Meyer, Roy Remmen, Philippe Beutels

**Affiliations:** 1 Department of Epidemiology and Social Medicine, Research Unit of Medical Sociology and Health Policy, University of Antwerp, Antwerp, 2610, Belgium; 2 Division of Physiopharmacology, University of Antwerp, Antwerp, 2610, Belgium; 3 Department of Primary and Interdisciplinary Care, University of Antwerp, Antwerp, 2610, Belgium; 4 Centre for Health Economics Research and Modelling Infectious Diseases (CHERMID), Vaccine and Infectious Disease Institute (VAXINFECTIO), University of Antwerp, Antwerp, 2610, Belgium; 5 School of Public Health and Community Medicine, The University of New South Wales, Sydney, Australia

## Problem statement

In Belgium, the average annual growth rate of per capita health spending decreased from 2.3% between 2000 and 2006 to 1.6% between 2007 and 2013 [1]. Nonetheless, Belgium's Gross Domestic Product (GDP) grew at markedly lower rates (2.1% and 0.8%, respectively), implying that pressures on sustaining affordable and equitable health care increase.

## Objectives

We assessed the impact of co-payment regulations on prices and sales figures for atorvastatin in Belgium before and after the patent expiry in 2013.

Policies targeted: Reference pricing system; Copayment regulations.

Region covered: EURO: Belgium (national)

## Methods

We related sales figures to coinciding price evolutions, and broke the costs down by their bearer. On the one hand we analysed a data extraction from IMS Health for Belgium for the period 2007-2013. The IMS Health database contains sales figures of a representative sample of community pharmacies including the number of packages sold (per CNK number) per month. On the other hand, we studied the corresponding unit price tables of the Belgian centre for Pharmaco-therapeutic Information (BCFI, an independent source).

## Results

In March 2013, the public price for Lipitor® 98*80 mg was EUR 123.51, and the price for Atorvastatin Mylan® (same package size) was EUR 62.52. The co-payment amount borne by patients was the same for these products (EUR 14.5 for each), but the costs borne by the government through the National Institute for Health and Disability Insurance (NIHDI) were markedly different (i.e. EUR 109.1 for Lipitor and EUR 48.02 for the generic Atorvastatin). For the 19,777 reimbursed packages of brand atorvastatin in 2013, the latter (reimbursed) costs amounted to EUR 2,157,671, which is about 1 million EURO more than would have been required to cover the same sales volume of the equivalent generic. Both packages were considered low-cost medicines resulting in the same 'minimized' patient's contribution, thereby eliminating any incentive for the physician or patient to choose a generic medicine.

## Conclusions

In this case, the reference pricing system was a vain attempt to curb public drug expenditures. Looking ahead at the patent expiry of rosuvastatin in 2016, the effectiveness of existing regulations to curb growing pharmaceutical expenditures requires urgent reconsideration, based on the lessons learnt from case studies such as ours. A potentially feasible option would be to abolish the maximum co-payment level per package in the Belgian reimbursement system for therapeutically interchangeable drugs.
